# Occupational injury and associated factors among cement factories workers in central Ethiopia

**DOI:** 10.1186/s12995-022-00348-0

**Published:** 2022-03-28

**Authors:** Demissu Seyoum Fresenbet, Ararso Tafese Olana, Abera Shibiru Tulu, Kababa Temesgen Danusa

**Affiliations:** 1 Departments of Public Health, College of Medicine and Health Science, Ambo University, Ambo, Ethiopia; 2 Departments of Midwifery, College of Medicine and Health Science, Ambo University, Ambo, Ethiopia

## Abstract

**Background:**

The cement industry experiences accident rate is high compared to other manufacturing industries. Hence, this study was aimed to assess the occupational injury and associated factors among cement factories workers in West Shoa Zone, Oromia regional state, Ethiopia 2020.

**Methods:**

An institution-based cross-sectional study design was employed. A total of 374 study participants were selected by using a systematic random sampling technique with K-interval. The collected data were entered into Epi-Info version 7 and analyzed by using SPSS version 21. Bivariate and multivariable binary logistic regression was used to identify the magnitude of occupational injury and the factor associated. A statistically significant association was declared at a 95% confidence interval and *p*-value<0.05.

**Results:**

The study revealed that the magnitude of occupational injury among workers of cement factories was 183(48.9%). Workers found in an engineering department were 3.7 times more likely to be injured than those who were working in a clinker department [AOR: 3.72, 95% CI: 1.35-10.18]. Those workers who were working room did not have danger signs were 2.9 times more likely to be injured than their counterparts [AOR 2.99, 95% CI: 1.79-4.98). Those workers who did not use personal protective equipment were 3.7 times more likely to be injured than those who were used Personal Protective Equipments [AOR: 3.78, 95% CI: 1.76 - 8.12].

**Conclusion:**

The magnitude of occupational injury in cement factories in the study area was high. The factories’ managers must provide adequate and quality safety materials in a timely and supervise their appropriate use and put danger signs where it is required.

## Introduction

According to International Labor Organization (ILO) report, annually over 2.3 million fatal and 313 million nonfatal occupational injuries were caused by occupational accidents and work-related diseases of which over 350,000 are caused by occupational accidents [1, 2]. Two-third of deaths from work-related diseases were 35% from cardiovascular and circulatory diseases, 29% from cancer, followed by occupational injuries 15% and infectious diseases 10% [3].

Occupational risk factors are responsible for 8.8% of the global burden of mortality due to unintentional injuries and 8.1% of Disability Adjusted Life Years (DALYs) due to this outcome. Under-reporting is a major challenge typically associated with occupational accidents and diseases statistics. This causes small resources to be allocated to preventive work, which has a negative result on the safety and welfare of the workers, the productivity of the factory, and the availability of the workers, moreover in countries with a non-favorable demographic situation [4, 5]. Multiple risk factors cause work-related injuries. The common factors in the workplace were psychosocial factors, ergonomic factors, socio-demographic characteristics of workers, and environmental and social conditions [6, 7].

The epidemiology of work-related injuries, in Ethiopia, is lacking due to scarce national and local data. proclamation No.377/2003, Ministry of Labor and Social Affairs (MOLSA) of Ethiopia is the responsible body to supervise labor administration, labor conditions, occupational safety, and health [8]. Skin allergies, eye irritation, and other work-related injuries are caused by cement dust and other occupational hazards worldwide [9].

The associated factors are job category, work experience, use of PPE, availability of PPE, health and safety training, hours worked per day (average), workplace supervision, work time (night, day, morning, afternoon), presence of danger sign, department, type of contract (permanent, temporary), hours worked per week, heat, and noise splintering objects, excessive dust, radiation, inadequate light, less/no ventilated rooms’,fire-extinguishers, presence /absence of emergency exit and around 4 % of the world’s gross domestic product (GDP) is lost annually in direct and indirect costs due to occupational accidents and work-related diseases [10]. Physical factors like workplace compliance, health status, body mass index, stress at work, body postures, active and enough breaks during work, and body condition at the end of work have a significant association with work-related disorders [11]. A study in japan indicates that, among never-smoking workers, The odds of occupational injury were high when regularly exposed to passive smoking at work or home, in comparison to never smoking men who were never exposed to passive smoking either at work or home [12].

Occupational accidents kill nearly 1000 people daily. There were over 313 million non-fatal occupational accidents that is about 860,000 people are injured daily. Annually, over 270 million occupational accidents occur that cause two million deaths according to International Labor Organization [13, 14]. Occupational injuries result from lack of personal protective equipment, lack of work experience, khat chewing [15]. A study done in Cairo indicated that cement workers and controls were matched for age, gender and smoking status. Chronic cough and expectoration were significantly higher among the exposed group [16].

Different investigations reported, in Africa, that the cement industry is a cause for many types of injuries mainly burn of a different part of the body, fracture, falling and hospitalization, high cost for treatment, and loss of productivity [17]. Sub-Saharan Africa appears to have the greatest rate per worker of occupational injuries followed by Asia (excluding China and India) [18]. The most commonly known hazard by respondents was cement dust which was known by almost 8 out of 10 respondents followed by noise (31.2%). About 20% of the respondents had suffered injuries while at work and 17% had been absent from work on account of work-related injuries in the last 1 year preceding the study [19]. In Zimbabwe, the magnitude of occupational injuries remains highly under-reported and there is no information on the number of occupational accidents in cement industries [20].

There is inadequate information on the gaps and needs of occupational health services in cement factories in Ethiopia. There is a great difference in studies explained above concerning the associated factors of occupational injuries in different industries in Ethiopia. Therefore, the aver all aim of this study was to assess the occupational injury and associated factors among cement factories workers in West Whoa Zone, Oromia regional state, Ethiopia 2020.

## Methods

### Study area and period

West Shoa Zonal capital Ambo is located at a distance of 114 km in the western direction from Addis Ababa. It has a total population of 2,607,827 of which 1,303,814 males and 1,304,013 females. There are 03 cement factories 88 Health centers, one General, six districts, and one referral Hospitals. The study was conducted in 02 cement factories (Dangote and Habesha cement factories). Totally 3743 workers were found in two cement factories (2003 workers are found in Habesha and 1740 workers in Dangote cement enterprise). The study was conducted from October 15 – December 15, 2020, in cement factories in the West Shoa zone Oromia regional state. The study design for this study was an institution-based cross-sectional study conducted among cement factories in central Ethiopia.

### Participants

In this study, all workers in cement factories were the source of the population. But, a randomly selected worker from a production process of cement factories, who gave information during the data collection period was the study population. Inclusion criteria were employees, who were directly engaged in the production process during the study Period & who had been working at least for one year in the selected factories were included in the study. But, exclusion criteria were those who were less than 18 years of workers, absent more than three times visit at the time of data collection and those who cannot answer due to hearing problems, administrative and supportive staffs were not included in the study.

### Sample size determination and sampling technique

The sample size was calculated by using single population proportion formula based on the following assumption by taking a proportion of occupational injury 10.4% [21], 95% confidence interval, and level of precision 4 and 10% non-response rate. Since the study population is less than 10,000, a correction formula was done. The sample for the second objective was calculated by using two population proportion formula and it was calculated through EPI Info version 7 statistical software package with the assumption of confidence level 95% (Zα/2 = 1.96), power 80% (Zβ= 0.84),10% non-response rate. The largest sample size from this was 346 which is equal 381 after adding a 10% non-response rate. But by the single population proportion formula, the sample size was 227 after a 10% non-response rate was added. Therefore, the final sample sizes were 381 cement workers. The study participants were selected from both factories by using a systematic sampling technique from the sampling frame using k interval. k = 3743/381 = 10.Then we select one starting number by choosing from 1 to 10 by lottery method. Then every kth respondents were selected from their registration up to the required samples were obtained.

### Data collection procedures

Data was collected through face-to-face interview by structured questionnaires majority of which adapted from occupational injury statistics and different relevant literature on occupational injury [15, 21]. The questionnaire focused on socio-demographic, behavioral, and environmental variables and occupational injury characteristics. A pre-test was taken place and the questionnaire was prepared in English, Amharic, and Afan Oromo and retranslated to English by a language expert to ensure its consistency. Six health professionals have participated (four data collectors, two supervisors).

### Variables and operational definitions

The dependent variable is Occupational injury and the Independent variables are Socio-demographic factors, Behavioral factors, and Working environment. Occupational Injury is an injury that will sustain on worker about the performance of his or her work in a cement factory within one year that causes hospitalization or working days lost or disabilities or death as reported by the worker or confirming clinical records or sick leave. Temporary absences from work of less than one day for medical treatment are not included [21, 22].

### Data processing and analysis

After data collection, data were checked for completeness, coded, and entered to Epi Info version 7, and was transported to SPSS version 21 for cleaning and analysis. Bivariate analysis was carried out to determine a significant association between each predictor variable and occupational injury with a *p*-value <0.2. Multivariate logistic regression was done for variables with a *p*-value less than 0.05 to control confounders. The backward model selection method was used to identify variables that remained for the final model. The goodness of fit model (Hosmer and Lemeshow) was used to select the best multivariate model. Variables with a *p*-value less than 0.05 at a 95% confidence interval in multivariate logistic regression were considered statistically significant. Standard error was used to check multi colinearity and confounding. The variables without multicollinearity were entered into a multivariate model. Finally, AOR with 95% CI, *p*-value <0.05 was considered as statistically significant.

### Data quality assurance

Data quality was assured during the data collection, recording, and analysis phase. The one-day training was given to data collectors and supervisors on the objective of the study, contents of the questionnaire, confidentiality, the right of respondents, and how to collect data. The pre-test on 5% of the sample was conducted at a branch of the Derba cement factory found in north Shoa. To identify the reliability of the data collection instruments and findings, data collectors and supervisors discussed the questionnaire so that the tool was modified for any inconsistencies and ambiguity before actual data collection.

### Ethics approval and consent to participate

The study was performed after obtaining ethical clearance from Ambo University, College of Medicine, and health sciences Institutional Review Board. Written informed consent was obtained from study participants after clearing up the objective and purpose of the study to every study participant.

## Results

### Socio-demographic characteristics of the respondents

A total of 374 (98.16%) respondents were included in the study and interviewed if they had experienced a work-related injury in the last year. Among 374 female respondents were only 66(17.6%). About 154(41.2%) of the respondents were in the age group 26-30 years. The majority 221(59.1%) of study participants were orthodox religion followers. Most of them about 149 (39.8%) of the respondents, learned Primary school.. Regarding the marital status of the respondents, the majority 238 (63.6%)of them were married. Among three hundred seventy-four study participants 289 (77.3%) were permanently employed (Table 1).

**Table 1:** Socio demographic characteristics of the respondents, Dangote and Habesha cement factories, West showa, December, 2020 (*n* = 374)

Characteristics	Frequency	Percent (%)
**Age**		
18-24	56	15.0
25-30	154	41.2
31-34	83	22.2
35-40	40	10.7
>41	41	11
**Sex**		
Male	308	82.4
Female	66	17.6
**Marital status**		
Married	238	63.6
Not married	136	36.4
**Salary**		
<1050	10	2.6
1050-1726	30	8
1727-3684	334	89
**Job category**		
Clinker	28	7.5
cement production	168	44.9
Raw material	71	19
Engineering	95	25.4
Other (Administrative staffs, Storage, transportation)	12	3.2
**Service year**		
<5 years	7	1.9
5-9 years	264	70.6
10-14 years	51	13.6
> = 15 years	52	13.9
**Employment pattern**		
permanent	289	77.3
Temporary	85	22.7
**Religion**		
Orthodox	221	59.1
Protestant	115	30.7
Muslim	12	3.2
Wakefata	26	7.0
**Educational status**		
Primary school(1-8) and below	149	39.8
secondary school(9-12)	55	14.7
Graduated by Diploma	80	21.4
First degree and above	90	24.1

### Occupational injury characteristics

**Fig. 1:**
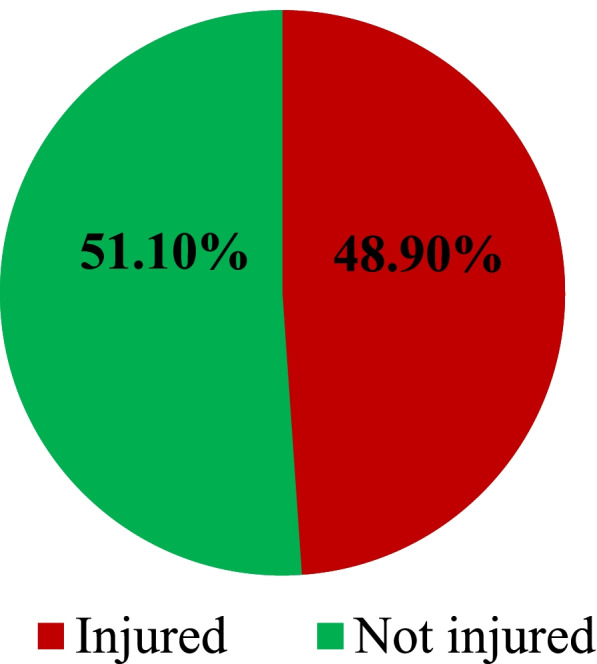
Presence of occupational injury among workers in Dangote and Habesha cement factories, West Shoa, 2020 (*n* = 374)

**Fig. 2:**
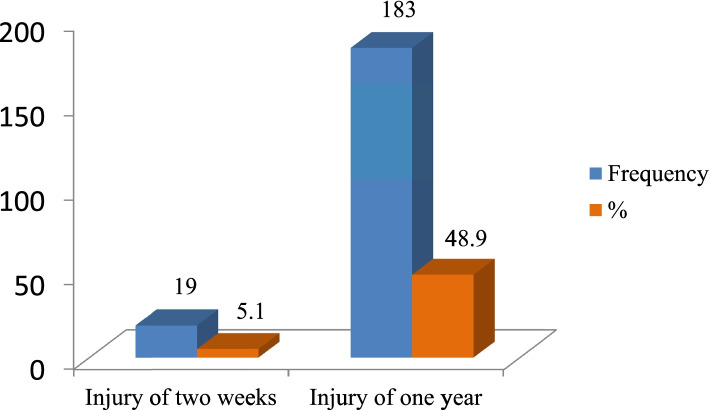
Distribution of occupational injury among workers in Dangote and Habesha cement factories, West Shoa, 2020 (*n* = 374)

A total of 183(48.9%) respondents were reported occupational injuries (OI) during the last 12 months (Fig. 1). One hundred thirty-three (35.6%) of the injured respondents reported had only sustained occupational injury once. The two-week prevalence was 19(5.1%) of these cases 12(3.2%) reported that they had sustained occupational injury once (Fig. 2).

The hand was the body part with the highest frequency of occupational injuries 77 (20.6%), Finger 50 (13.4%, Eye 36(9.6%) and toe 24 (6.4%), were other sites frequently affected. The main types of injuries reported were cuts 86(23%), abrasions 61(16.3%), eye injury 36(9.6%), electrocutions injury 32(8.6%) & fracture 29(7.8%) (Table 2).

**Table 2:** Distribution of occupational injury by part of the body affected and type of injury, Dangote and Habesha cement factories, west shoa, December, 2020 (*n* = 183)

Variables	Frequency	Percent
**Part of the body affected**		
Hand	77	20.6
Finger	50	13.4
Eye	36	9.6
Toe	24	6.4
Lower arm	21	5.6
Multi-location	21	5.6
Upper arm	20	5.3
Lower leg	20	5.3
Head	12	3.2
Hip	8	2.1
**Type of injury**		
Cut	86	23.0
Abrasion	61	16.3
2003Fracture	29	7.8
Puncture	24	6.4
Burn	12	3.2
Amputation	12	3.2

### Causes of injury

**Fig. 3:**
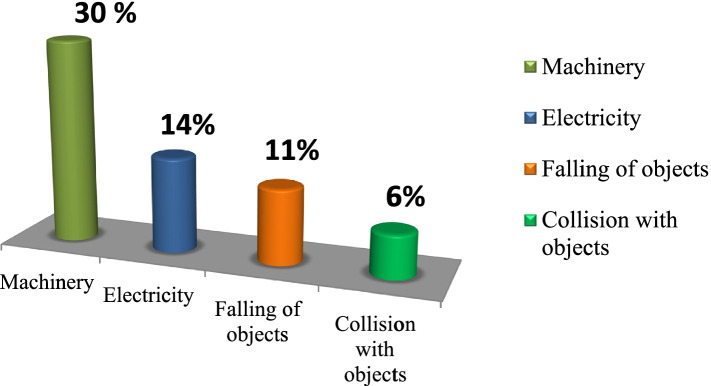
Causes of occupational injury among workers in Dangote and Habesha cement factories, West Shoa, 2020 (*n* = 374)

The most common agent stated as the cause was machinery 111(30%), electricity 52(14%), falling of objects 41(11%), hand tools and carrying heavy objects 32(9%), collision with objects 24(6%) followed by hot substances 20(5%) (Fig. 3).

Fifty (13.1%) of the occupational injuries occurred on Monday 50 (13%), on Wednesday 41(11%) followed by Thursday 36 (10%). The most common time of injury was in the afternoon 54(14%) followed by midnight 25(7%). About 50(13%) and 45(12%) of workers thought the reason for injury was due to thinking about private affairs and being new for the work process respectively (Table 3).

**Table 3:** Distribution of occupational injuries in the last 12 months by cause of injury, day of injury and time of injury among 183 injured respondents, dangote and habesha cement factories, december,2020 (*n* = 183)

Variable	Frequency	%	variable	Frequency	%
Reasons of Injury			Day of injury		
Being new for the work process	45	12	Monday	50	13
Thinking about private affairs	50	13	Tuesday	24	6
Due to other medical problems	24	6	Wednesday	41	11
Those think accident is beyond control	4	1	Thursday	36	10
Those think it was working behavior	36	10	Friday	13	4
**Causes of Injury**					
Machinery	111	30	Saturday	8	2
Hit by falling objects	41	11	Sunday	12	3
Electricity	52	14	morning	8	2
Hand tools	32	9	Afternoon	54	14
Hot substances	20	5	Evening	16	4
			Mid-night	25	7

### The severity of occupational injuries

Of the total 183 injured respondents, 84(46%) were hospitalized, 76(20%) accounting for hospitalization more than 24 h. One hundred fifty-three (41%) were absent from work for 2-6 days and 25(7%) were absent from work for 15-30 days (Table 4).

**Table 4:** Severity of occupational injuries as reported by the respondents andconfirmed by the investigator, Dangote and Habesha cement factories, December, 2020 (*n* = 183)

	Frequency	Percent
**Hospitalization**	84	46
How long (*n* = 84)		
<= 24 h	8	2
Greater than 24 h	76	20.3
**Working days lost**		
<2	5	1.3
2-6	153	41
15-30	25	7

### Work environment characteristics

Seventy-eight (20.9%) respondents worked for more than 48 h per week. One hundred sixty-one (43%) of the respondents reported that their workplaces were not supervised regularly. About 116 (31%) of the respondents were not taken any health and safety training (Table 5).

**Table 5:** Work environment characteristics of respondents in dangote and habesha cement factories, December, 2020 (*n* = 374)

Variable	Frequency	Percent
Working hour in a week		
Less than 8 h	77	20.6
8 h	214	57
Greater than 8 h	83	22
Safety training taken place	258	69
Manual handling	204	55
Safety supervision taken place	213	575

### Behavioral characteristics

Ninety-four (25%) and 47(13%) of the respondents consumed alcohol and smoked cigarettes respectively. Most 61(16%) consumed alcohol 1-3 days per week. Forty-four (12%) of the respondents reported that they worked continuously for more than eight hours. About 4 (12%) of the respondents worked at night. Eighty-two (22%) of the respondents respond there were no warning signs in their workplace. One hundred eleven (30%) of the respondents were not used personal protective equipment at their workplace. All the study participants were interviewed for non-use of protective equipment and reported the most frequent reasons were lack of personal protective equipment 81(22%), and not knowing how to use 19 (5%) (Table 6).

**Table 6:** Behavioral characteristics of respondents in Dangote and Habesha cement factories, December, 2020 (*N* = 374)

Variable	Frequency	Percent
Cigarette smoker	47	13
**How much**		
less than one pack every day	17	5
1-2 pack/days	29	8
Alcohol drinker	94	25
**How often**		
Every day	37	10
1-3 days/ week	61	16
Occasionally	4	1
Chewing chat	39	10
**Reason for sleeping disorder**		
Working greater than 8 h	28	8
Working at the night	44	12
Working greater than one areas	9	2
Don’t use PPE	59	16
**Reason for not using PPE**		
PPEs are not comfortable	17	5
Employer doesn’t provide	81	22
Don’t know how to use	19	5
PPEs reduce performance	12	3
PPEs expose to another accident	4	1

### Factors associated with injury in the cement factories

A multivariate binary logistic regression analysis was done to identify independently associated variables with injury in cement factories. The multivariate binary logistic regression analysis revealed that the following factors had an association with injury in cement factory: Job Category, Presence of danger sign in the working room, Cigarette smoking, Provision of PPE, educational status of the workers, and using PPE of workers were statistically significant factors at *p*-value <0.05. Workers found in an engineering department were 3.7 times more likely to be injured than those who were working in a clinker area [AOR: 3.720, 95% CI: 1.358-10.188]. Workers who had learned up to primary school were 1.8 times more likely to be injured than those who have a first degree and above. Those workers who were working in where a working room that did not have danger signs were 2.9 times more likely to experience an injury than those who did have a danger sign in the working room [AOR 2.996, 95% CI: 1.799-4.989). Workers who smoked cigarettes were 1.7 times more likely to be injured than their counterparts [AOR: 1.730, 95%CI: 1.030-2.907]. Those workers who did not use PPE were 3.7 times more likely to be injured than those who were used PPE [AOR: 3.788, 95% CI: 1.766 - 8.124] (Table 7).

**Table 7:** Bivariate and multivariate analysis for factors associated with magnitude of injury in cement factory among workers in West Shoa Zone, Oromia 2020

Variables	Category	Injury		COR(95%CI)	AOR(95%CI)	***P-Value***
		Yes *N* = 183	No *N* = 191			
Job Category	Clinker	18 (4.8%)	10 (2.7%)	1.00	1.00	
	Cement Production	109 (29.1%)	59 (15.8%)	0.974 (0.423, 2.247)	0.973 (0.380, 2.488)	0.954
	Raw Material	33 (8.8%)	38 (10.2%)	2.073 (0.840, 5.112)	2.201 (0.815, 5.948)	0.120
	Engineering	22 (5.9%)	73 (19.5%)	5.973 (2.409, 14.811)	3.72 (1.358, 10.188)	0.044*
Education Status	Primary school(1-8)	67 (17.9%)	82 (21.9%)	1.753 (1.032, 2.977)	1.897 (1.006, 3.579)	0.048*
	Secondary school(9-12)	26 (7%)	29 (7.8%)	1.598 (0.813, 3.140)	1.418 (0.621, 3.241)	0.407
	Graduated by diploma	37 (9.9%)	43 (11.5%)	1.665 (0.906,3.057)	1.316 (0.645, 2.684)	0.451
	First degree and above	53 (14.2%)	37 (9.9%)	1.00	1.00	
Provision of PPE	Yes	119 (31.8%)	98 (26.2%)	1.00	1.00	
	No	64 (17.1%)	93 (24.9%)	1.765(1.164, 2.674)	0.500 (0.245, 1.020)	0.057
Presence of danger sign in working	Yes	94 (25.1%)	44 (11.8%)	1.00	1.00	
	No	89 (23.8%)	147 (39.3%)	3.529 (2.263,5.503)	2.996 (1.799, 4.989)	0.001*
Cigarette smoking	Yes	74 (19.8%)	127 (34%)	2.923 (1.918, 4.454)	1.730 (1.030, 2.907)	0.038*
	No	109 (29.1%)	64 (17.1%)	1.00	1.00	
Using PPE of workers	Yes	100 (26.7%)	51 (13.6%)	1.00	1.00	
	No	83 (22.2%)	140 (37%)	3.307 (2.145, 5.099)	3.788 (1.766, 8.124)	0.001*

Key, 1.00: Reference Category, *AOR* Adjusted odd Ratio, *CI* confidence interval: * = indicates significant association at *p* < 0.05. *COR* Crude Odd Ratio

## Discussion

This study assessed the prevalence of injury in cement factories among workers in 12 months was 183(48.9%). The study’s most common agent stated as the cause of occupational injuries was machinery 111 (29.7%), Electricity 52(13.9%) and Hit by falling objects 41(11%) followed by hand tools 32 (8.6%). This could be due to unguarded machine parts and non-use of PPE. The finding is lower compared with a study conducted in Arba Minch town southern Ethiopia 80.8% [2], and in Mekelle city, Ethiopia 58.2% [23]. This discrepancy might be due to differences in socio-demographic characteristics of participants, setting, study design, and study year. It also might be due inclusion of minor injuries such as scratches in the study conducted in Arba Minch Town. This prevalence is higher than the study conducted in Mugher cement factory 10.4%(21),northern Gondar, Ethiopia 33.5% [3] and Addis Ababa 38.5% [15]. This may be due to unfamiliarity with work processes and exposures and lack of safety training and non-availability and non-practice of Safety measures [5].

The Ministry of Labor and Social Affairs of Ethiopia, with Regional Offices, provides inspection services and expert advices on Occupational Safety and Health. Standard inspections are made based on priority hazards. The employer is obliged by law to implement the experts’ advice upon receiving the inspectors’ notification. Regular measurements of hazards such as industrial noise and dust are rarely done due to lack of training, lack of skill in measurement and non-existent instrumentation at the factory level. This happens to be the case although employers have the responsibility to let workers know and that the employees have the right to know their exposure level by law [8].

In this study job category of the workers was associated with injury occurrence. Workers working in the engineering department were 3.7 times more injured than those working in the clinker which is consistent with a study done in North Gondar3]. This may be due to most of the workers in engineering are from a high level of education which might make them overconfident and so not using PPE like in a study done in Bangladesh [6].

The presence or absence of danger signs in the working room was significantly associated with a work-related injury. Workers who worked where danger sign (safety rules) were not present in the working room was 2.9 times more likely to be injured than those who work in the presence of danger sign in the working room which is similar to the study done in large scale metal manufacturing industries in Ethiopia [7]. This might make them not give focus on the machines and other uncovered equipment that could harm them.

Cigarette smoking was significantly associated with a work-related injury. Workers those smoke cigarettes were 1.7 times more likely to be injured than those who did not smoke. The possible reason might be workers who were addicted to substances mainly took those substances in the afternoon. But the working condition was not suitable so the majority were in non-moody condition. This finding is consistent with findings in Ethiopia [7], Mizan Aman, South Ethiopia [9] but inconsistent with findings in Japan [12] and south India [11]. The difference between these findings might be, due to the difference in temperature which pushes them to be addicted to cigarettes.

Using or not using personal protective equipment of workers was also significantly associated with a work-related injury. Those workers who did not use full PPE regularly were 3.78 times more likely to be injured than their counterparts. This is consistent with the study done in Ethioiap [10, 13, 15].

### Limitation of the study

This study is cross-sectional so it does not show the temporal relationship and the possibility of recall bias may result in under-reporting and misreporting of events. In addition, there is a lack of studies in occupational injury in cement factory particularly in Ethiopia to associate other studies.

## Conclusion and recommendations

This study showed that the prevalence of occupational injuries was 48.9%. This finding shows that occupational injury in cement factories is higher. The major reasons for injury were job category, education level, workers’ behavior, absence of danger signs in the working room, and lack of PPE provision. To improve occupational injuries, Preventive measures like Provision, supervision of adequate and high quality personal protective equipment timely and their appropriate usage should be needed. Additional further study, which includes qualitative research study, should be needed in the future.

## Data Availability

The datasets used and/or analyzed during the current study are available from the corresponding author upon request.

## Abbreviations

AOR: Adjusted Odd Ratios
CI: Confidence Interval
DALY: Disability Adjusted Life Years
ILO: International Labor Organization
MOLSA: Ministry of Labor and Social Affairs
OI: Occupational injuries
PPE: Personal Protective Equipment
WHO: World health organization
